# ‘Beyond the Scale’: A Qualitative Exploration of the Impact of Weight Stigma Experienced by Patients With Obesity in General Practice

**DOI:** 10.1111/hex.14098

**Published:** 2024-06-11

**Authors:** Leona Ryan, Fiona Quigley, Susie Birney, Michael Crotty, Owen Conlan, Jane C. Walsh

**Affiliations:** ^1^ School of Psychology University of Galway Galway Ireland; ^2^ School of Communication and Media Ulster University Belfast Northern Ireland; ^3^ Irish Coalition for People Living with Obesity (ICPO) Dublin Ireland; ^4^ Best Weight Clinic Dublin Ireland; ^5^ School of Computer Science and Statistics Trinity College Dublin Dublin Ireland

**Keywords:** general practice, obesity, patient–provider, qualitative, weight stigma

## Abstract

**Objective:**

Obesity is a complex, chronic, relapsing disease that requires an individualised approach to treatment. However, weight stigma (WS) experienced in healthcare settings poses a significant barrier to achieving person‐centred care for obesity. Understanding the experiences of people living with obesity (PwO) can inform interventions to reduce WS and optimise patient outcomes. This study explores how patients with obesity perceive WS in general practice settings; its impact on their psychological well‐being and health behaviours, and the patients suggestions for mitigating it.

**Methods:**

In‐depth semistructured interviews were conducted with 11 PwO who had experienced WS in general practice settings in Ireland. The interviews were conducted online via Zoom between May and August 2023; interviews lasted between 31 and 63 min (*M* = 34.36 min). Interviews were audio‐recorded, transcribed verbatim and analysed using inductive reflexive thematic analysis.

**Results:**

Three overarching themes specific to participants' experience of WS in general practice were generated: (1) shame, blame and ‘failure’; (2) eat less, move more—the go‐to treatment; (3) worthiness tied to compliance. A fourth theme: (4) the desire for a considered approach, outlines the participants' suggestions for reducing WS by improving the quality of patient–provider interactions in general practice.

**Conclusion:**

The findings call for a paradigm shift in the management of obesity in general practice: emphasising training for GPs in weight‐sensitive communication and promoting respectful, collaborative, and individualised care to reduce WS and improve outcomes for people with obesity.

**Patient or Public Contribution:**

PPI collaborators played an active and equal role in shaping the research, contributing to the development of the research questions, refining the interview schedule, identifying key themes in the data, and granting final approval to the submitted and published version of the study.

**Abbreviations**
5As: Ask, Assess, Advise, Agree, Assist
BMI: Body Mass Index
GP: General Practitioner
ICPO: Irish Coalition for People living with Obesity
PPI: Patient and public involvement
PwO: Person/patient with obesity

## 1 Introduction

Obesity is known as a complex, chronic and relapsing disease characterised by the accumulation of excess adiposity, which is associated with different degrees of morbidity and mortality [1]. Excess adiposity elevates the risk of health complications including cardiovascular disease, metabolic dysfunction (Type 2 diabetes, nonfat liver disease, hypertension) and cancers [2]. It also increases the risk of functional limitations and psychological complications, including anxiety, depression, and mood disorders [3, 4].

Our understanding of the aetiology and pathophysiology of obesity has grown considerably in the last decade, moving away from oversimplified energy expenditure, toward recognition of the complex interplay of genetic, behavioural, psychological and environmental factors that contribute to its onset and maintenance [3, 5]. Yet, as individuals differ in body composition, fat distribution, and how the body uses fat, the amount of excess adiposity that is symptomatic of impaired health varies considerably [6, 7, 8]. Given the complexity and heterogeneity of the disease, an individualised, person‐centred approach to the management of obesity is essential. However, a known barrier to person‐centred care for obesity is weight stigma (WS) experienced in healthcare settings [9, 10].

WS is described as the devaluation and denigration of an individual or a group because of their body size [11]. There are three conceptualisations of WS: *experienced*, *anticipated* and *internalised* [12]. WS can be *experienced* when people with obesity (PwO) face prejudice and/or discriminatory actions toward them, this can be communicated overtly or more subtly through microaggressions [13]. PwO may come to *anticipate* future mistreatment arising from past experiences or cues that signal potential weight‐based discrimination [14]. *Internalised* stigma occurs when PwO apply and direct negative societal beliefs and biases about weight towards the self, leading to a negative self‐image, guilt, shame and low self‐esteem [15].

WS is pervasive in healthcare settings where substantial evidence supports its contribution to overall poor health outcomes and the maintenance of obesity via physiological [16], psychological and behavioural pathways [17]. In addition to physical ill health, WS is associated with poor psychosocial outcomes [11], and an increased risk of disordered eating intentions and behaviours [18, 19, 20].

Systematic review evidence indicates that stigmatising patient–provider interactions are the greatest driver of WS experienced in healthcare settings [13, 21]. While considerable research details the occurrence and prevalence of WS in healthcare settings [22, 23], less is understood about how stigmatising patient–provider interactions, particularly in general practice settings, impact PwO and their health behaviours [13, 24]. General practitioners (GPs) in primary healthcare settings have been identified as being best placed to initiate person‐centred care for obesity, given their long‐term relationship with patients [10, 25]. Thus, more research in this context is required to understand the potential interpersonal barriers to quality healthcare faced by PwO, and the role of GPs in perpetuating and deconstructing these barriers.

Several studies have examined the best method to reduce WS in healthcare settings [26, 27]. Most recently, a narrative synthesis of studies undertaken with healthcare students, trainees and professionals identified the most effective interventions to contain causal and controllability information, empathy evoking, having a weight‐inclusive approach and mixed methodology [28]. These findings are supported by an interprofessional systematic review and meta‐analysis investigating reduction strategies. However, results indicated marginal effects of these approaches and highlight the lack of longitudinal studies to quantify the lasting impact of the interventions [29]. Additionally, the extant research is predominantly theory driven and quantitatively measured, with the notable absence of the patient's lived experience to inform the development of a WS reduction strategy [24, 27, 30, 31, 32].

To achieve nonstigmatising person‐centred care for obesity, it is critical to understand how WS is experienced by PwO within patient–provider interactions, the impact it is perceived to have on their health, and the patients suggestions on how to reduce it. Considering this, the current study aimed to explore how WS is perceived by PwO in patient–provider interactions in general practice. The study aimed to explore the impact if any, on psychological and behavioural implications of the same. Finally, solutions to reducing weight‐stigmatising experiences in GP consultations informed by the perspective of PwO are presented.

## 2 Methods

The reporting of the study was guided by the Consolidated Criteria for Reporting Qualitative Research (COREQ) [33]. After critically reflecting on the COREQ reporting guidelines, it was determined that certain elements did not align with our analytical method, reflexive thematic analysis (RTA). The component ‘data saturation’, was not compatible with the assumptions or methodological approach that underpins RTA. To retain the fidelity of the methodological approach taken, ‘data saturation’ is replaced with ‘information power’ where the collection of rich, meaningful data is prioritised over quantity of respondents [34, 35]. Additionally, participant validation was deemed impractical due to the researcher's central role in data interpretation and the potential for participant perspectives to change over time [36].

### 2.1 Research Team and Reflexivity

The research team is composed of authors with backgrounds in patient advocacy (S.B.), medicine (M.C.), healthcare communication (F.Q.), computer science (O.C.) and health psychology (J.W., L.R.), one of whom has subject area expertise (L.R.). Reflexivity requires the authors to take into account the potential impact their personal stance and beliefs related to the phenomenon being explored may have on the study's design, data collection, data analysis, and interpretation of findings [30]. To promote transparency, preconceptions were documented in reflexive journals and, through a process of constant comparison, they were critically reflected on and addressed by the team at every stage of the analysis.

### 2.2 Public and Patient Involvement (PPI)

The study forms part of a larger project aiming to build the evidence base for an obesity education and training tool for GPs. PPI collaborators were invited onto the project to represent the needs and wants of the knowledge beneficiaries throughout the process. In this instance, PPI is not an external advisory panel; instead, they are integral members of a collaborative team that includes researchers, an expert by lived experience, and a GP specialising in obesity medicine. The PPI collaborators played an active and equal role in shaping the research, contributing to the development of the research questions, refining the interview schedule, generating key themes in the data, and granting final approval to the submitted and published version of the study.

### 2.3 Study Design

In the current study, we adopted a qualitative interpretivist paradigm with a constructionist epistemology to underpin the methodological approach used to collate and analyse the collected data [34]. The framework reflects our philosophical assumption that reality is subjective, thus it is multifaceted and is socially constructed through human interactions [37]. This was considered an appropriate lens through which to explore WS in the context of obesity management in general practice from the perspective of patients with obesity, as it privileged participant insights' while simultaneously acknowledging the reflexive influence of the researchers' role in interpreting the analysis [38]. Ethical approval for the study was granted by the University of Galway Research Ethics Committee (approval reference number: 2022.10.014).

### 2.4 Participant Selection and Participant Information

Participants were recruited through purposive and snowball sampling. The Irish Coalition for People living with Obesity (ICPO) and The Best Weight Clinic, Dublin, Ireland aided study recruitment by providing a brief description of it to their members and patients. Additionally, a recruitment campaign was published on social media (*X*) to communicate the study to a wider audience. Interested participants contacted the lead author and were emailed study information packs (study overview, data protection procedures and consent forms). Those that met the study inclusion criteria—aged 18 years and older, living with obesity (operationalised as body mass index (BMI) of >30 kg/m^2^), and as having experience of general practice services in Ireland—were invited to participate. Written informed consent was collected from the participants before the commencement of the interviews. No reasons for refusing to participate were disclosed or recorded. In total, 11 participants took part in the study. The study consisted of 10 female participants, and 1 male participant, ranging in age from 19 to 72 years old (*M* = 39.27, SD = 15.49). Participant characteristics are shown in Table 1.

**Table 1:** Participant characteristics.

Sex	Age	Urban/rural^a^
F	19	Rural
F	27	Rural
M	72	Urban
F	36	Urban
F	30	Rural
F	55	Urban
F	49	Urban
F	50	Urban
F	31	Rural
F	35	Rural
F	28	Rural

a Irish urban/rural classification: Urban: population > 1500; Rural: population < 1500 [39].

### 2.5 Data Collection

Individual semistructured interviews were conducted online via Zoom between May and August 2023; interviews lasted between 31 and 63 min (*M* = 34.36 min). The interview guide was informed by data collected through a qualitative evidence synthesis exploring the experiences and perceptions of PwO while navigating the healthcare system [13], and the broader literature related to the study (Supporting Information S1: Table S1). It was refined after piloting with PPI collaborators. The guide was used adaptively to facilitate a conversational flow, keeping in mind the sensitive nature of the topic under exploration. For consistency, one author (L.R.) conducted the interviews. Comprehensive field notes were recorded after each interview to document observations, initial interpretations, and contextual details to enrich the data analysis. The interviews were digitally recorded on Zoom with the participants' consent and transcribed verbatim by the lead author before being imported into NVivo 20 [40] software for analysis.

### 2.6 Data Analysis

Reflective thematic analysis [34] was chosen for the current research due its capacity for multilayered data analysis. This method integrates both semantic coding, which prioritises the surface‐level meaning ascribed by participants, and latent coding, which delves deeper to uncover implicit themes and interpretations. This comprehensive approach to qualitative data analysis allows for a nuanced understanding of participant experiences while acknowledging the potential influence of broader social and cultural contexts. In practice, this was a nonlinear process that moved back and forth between the six phases of the analytical approach outlined by Braun and Clarke [34]. The lead author familiarised herself with the data by reading and re‐reading the transcripts. Orthographical notes were taken to contextualise meaningful patterns identified across the collected data. Inductive coding at both the semantic level and latent level was conducted, iteratively refined and organised into meaningful clusters. To uphold methodological rigour, the research team engaged in structured reflexive discussions [41, 42] at each stage of the analysis. This collaborative approach ensured that the interpretations of one author were not privileged in the reporting of the analysis. This practice facilitated the consolidation of codes into thematic patterns reflecting shared meaning, ultimately generating candidate themes and subthemes. To ensure congruency and coherence, the team reviewed the themes and subthemes in light of the research question at the individual code level, and at the level of meaning captured across the entire data set. This iterative process led to theme refinement and finalisation. Semantic and latent themes and subthemes were generated through this process and are presented below.

## 3 Results

Four themes were generated, ‘Shame, blame and failure’, ‘Eat less, move more—the go‐to treatment’, ‘Worthiness tied to compliance’ and ‘The desire for a considered approach to obesity management’. The findings are supported by multiple extracts to contextualise the patient experience of WS in general practice settings.

### 3.1 Shame, Blame and, ‘Failure’

This theme encapsulated the pervasive experience of WS experienced by PwO through interpersonal interactions with their GP. WS was primarily expressed through an ‘insensitive approach’ to the initiation of discussion related to weight and weight‐related health concerns. These experiences were perceived to negatively impact upon the participants self‐perception and attitude towards engagement.

Not being even met as a human being, you know. Not even asked, do I think I have an issue with my weight? Do I think I'm overweight? Maybe I'm the happiest camper in the world and I'm perfectly fine, or maybe not. But there was no room for any of that because of the manner in which it was just blurted out, to the point of me personally just shutting down.

Shame was described as a ‘tool’ doctors employed to ‘motivate’ weight loss with inferences made that it was within the participants volitional control to do so. Failure to lose weight was perceived to reflect poorly on the participants personal characteristics. In particular, there was an overarching sentiment that GPs viewed obesity as manifest of ‘laziness’, ‘greed’ and related to ‘low intelligence’. These negative attributions appeared to increase in intensity and frequency the higher the patients weight.

When I returned with the same issue, struggling to lose weight, he [GP] was so patronizing and made me feel like I was stupid for not understanding ‘eat less, move more’, or that I was lying.

This was strongly inferred by the participants' when their GP used a portion plate to illustrate to patients the amount of food to eat. This was perceived to be condescending and dismissive of their attempts to explain that eating less was not working for them. This conveyance of stigma created an environment of distrust, further alienating participants from seeking treatment.

My regular doctor would be, do you know [sic], he'd get out the portion plate. And he'd be like this is your portion plate, this is what you eat. And you'd be like, okay…. I understand that. But I was just like, it's not working for me, there's something going on with me!

The communication of stigma was not reserved only for spoken words; participants described the common practice of being referred to as a ‘failure’ in both oral and written communication between their GP and other healthcare professionals: ‘But when you read: ‘I saw a morbidly obese, forty‐nine year old woman today, [patients name] is an Ozempic failure’, it does something to you. It hurts you know. Words matter’. The internalisation of the concept of being a ‘failure’ had a profound impact on participants' psychological well‐being and coloured how they related to themselves and to others: ‘Failure, blame, shame, they're all kind of words that would be left there [sic], it's like me now regaining, I'm thinking I'm failing the people who have helped me, because I'm regaining’.

It was my fault you know. And it's all about blame and shame. Those two words you know [sic] and failure. You failed the diet, you failed all these different things you know [sic]. So that's what it was about with my GP all the time, pretty much.

There was a sense that participants were left alone to navigate their general health concerns. Participants recounted their experiences of having nonweight related health complications blamed on their weight in the absence of investigations into more plausible or alternative causal factors. For example, one participant described having her weight maligned as the reason for a miscarriage: ‘I've had several miscarriages, and one of them I miscarried at 14 weeks. And as I was miscarrying, I was told the next time if you do want to get pregnant you really should lose weight before you get pregnant again. Because you'll have a better chance of the baby surviving. This is, as I am being told I am miscarrying so, that was lovely’.

Paradoxically, cases of GPs using inappropriate humour to address weight in clinical encounters permeated the data set. This finding oscillated between descriptions of GPs avoiding the subject of weight in spite of the patient wanting to address it in the consultation: 'Some doctors will be like, ‘oh sure you know yourself eat less move more. Sure, I should be doing it myself’, and they'd be laughing. They have a fear about talking about people who are overweight. Where you're like [sic], no I am overweight, but like [sic], but what are we going to do about it?’. These particular encounters brought the question of intentional WS by GPs into consideration for the participants, with some theorising that it was a lack of education and awareness that underpinned some GPs' poor interpersonal interactions.

Oh absolutely, it is reflective of more education needed in the area [of obesity].

### 3.2 Eat less, Move More—The Go‐To Treatment

In general, participants reported that they did not feel supported in managing their obesity. The dominant clinical approach practiced by GPs was to advise patients to eat less and move more. Participants described how this advice was oftentimes given with no direction of ‘how to eat, or in what way to move’. Echoing the previous theme, there was a perceived lack of support for alternative weight management options even when it was apparent that the advised lifestyle approach was not working for the participants.

I sat in front of him and I told him, I said I would eat the table if you told me it was ok, right now I would eat that table in front of you. I said, I go to the gym three days a week, I walk any day that I'm not at the gym and I cannot stop eating, I can't stop myself and I lived on a program for 4 years, like a really successful program for 4 years. I said, I cannot stop myself. He turned around and he said to me, ‘you need to work harder in the gym, you're obviously not doing enough, you need to walk faster and you just have to stop eating and you'll be fine’. And from that day to this, I have put on three and a half stone.

BMI was bilaterally perceived to be a ‘misleading’ classification of health that subverted further investigation of the participants’ overall health and access to required, or preferred treatment. The dangers of relying on BMI as the ‘gold star’ indicator of health in the sample were multifaceted. For example, one participant recovering from bariatric surgery lost weight at a pace that was flagged by a practice nurse as warranting further investigation by the participants GP—who, guided by BMI, overlooked the weight of excess skin and advised her to ‘keep losing weight’; she was living with an undiagnosed eating disorder at that time. In the broader context, BMI stratifications were identified as representing a barrier for participants in acquiring health insurance and accessing preferred obesity treatment: ‘In relation to qualifying for bariatric surgery, it's all down to your BMI, not the fact you may have sleep apnoea, fatty liver or, you know [sic], high blood pressure, or diabetes, that isn't taken into consideration, it's your BMI, if you don't meet a certain BMI—forget about it’.

A trend that weaved through participants' narrations was the perception that GPs were reluctant to investigate *why* weight loss was not being achieved through traditional methods. Specifically, in the female sample, participants reported that concerns about hormonal disorders in relation to weight gain were undermined and dismissed by their GPs, oftentimes resulting in them suffering with additional symptoms years before receiving a diagnosis.

My regular doctor would […] get out the portion plate. And he'd be like this is your portion plate, this is what you eat. And you'd [sic] be like, okay I understand. But I was like it's not working for me, there's something going on with me! But I felt like it was just, the weight was an explanation for everything. It wasn't a side effect of what was actually going on for me. So I eventually, after six years of pushing, got diagnosed with polycystic ovary syndrome (PCOS).

### 3.3 Worthiness Tied to Compliance

This theme explored participants' beliefs about the lasting effects experiencing poor interpersonal interactions with their GP had on their self‐efficacy and their willingness to engage with their GP. There was an overarching sentiment that people with obesity are not ‘worthy of consideration’ when it came to having their health concerns addressed. These beliefs translated into patients anticipating differential healthcare treatment and being fearful of challenging the ‘status quo’. A nuanced finding highlights the perceived association made between the prescription of dismissive generalised lifestyle advice for weight management, internalised blame and shame and the adoption of disordered eating behaviours.

#### 3.3.1 Unworthy of Treatment

GPs' reliance on oversimplified approaches to manage obesity coupled with a perceived unwillingness to investigate alternative influences on weight gain, or prohibitors to weight loss, left patients feeling ‘unworthy’ of adequate healthcare. Participants generally expressed a despondency toward seeking healthcare, with one participant describing a significant physical response to the anticipation of receiving poor treatment from their GP: ‘I was so tense before I went in, I was terrified! I would throw up with fear because of what could be said to me’.

Participants described a reluctance to challenge their GP by questioning decisions regarding their health, in fear of it impacting upon their ability to access other healthcare services. Compacting this fear was a belief that as a person living with obesity, they were on the peripheral of the healthcare system; that a certain level of compliance was necessary for them to be considered ‘acceptable’ for treatment by their GP: ‘They're petrified to say the wrong thing, do the wrong thing, for it to impact their care. And [sic] you don't know if you're allowed to complain or not, because you're petrified if it will’.

And like [sic] I was doing my best, it was probably the healthiest I had ever been […] and it still wasn't enough for me to be treated correctly. For me to be accepted as a human who needed help, instead of a fat person who needed help. It wasn't enough for him.

#### 3.3.2 Oversimplified Advice May Have Unintended Consequences

Participants' recounted lifelong experiences of receiving dismissive, generalised lifestyle advice for weight management, often beginning in childhood. The prescription of generalised weight management, delivered in a judgemental manner, impacted how participants viewed themselves and, in some instances, affected their relationship with food. This was perceived to manifest where prior attempts at weight loss proved unsuccessful and subsequent consultations seeking explanation for the lack of progress yielded further dismissive, weight‐centric advice from GPs. These interactions compounded feelings of blame and shame, leading some to internalise their struggles with weight loss as evidence of personal failings. In turn, this prompted the adoption of restrictive and binge‐purge eating behaviours in some participants, with these behaviours persisting throughout their lives.

‘I understand that the language that was used with me is what put me down the path I'm on [disordered eating]’. And I do pin it down to that language. And [sic] that's what got me here; whereas [sic] if a different kind of language had been used with me at the time, that wouldn't have entered my mind frame.

### 3.4 The Desire for a Considered Approach to Obesity Management

The framing of weight‐based discourse was determined as having a significant influence on participants willingness to engage with their healthcare provider. Participants' cautioned on the use of judgemental or shaming language when communicating with patients with obesity. There was an expressed desire to have a more ‘considered approach’ to weight‐related concerns that were underscored by patients wanting to have their health concerns listened to, and understood. The importance of listening to patients without rushing to judgement was second only to calls for GPs to be mindful of the language they use to communicate about weight with their patients. The ramifications of pervasive disparaging commentary about weight had real‐life implications for the participants in the sample, which emphasises that ‘words matter’ in consultations with patients with obesity: ‘So basically, its language, think before you open your mouth or […] I used to do radio for years, the first line on the instructions was: engage brain before opening mouth’.

Because doctors were using the wrong measures and the wrong words to explain my state of health, because of that I went to fight something that wasn't there to be fought—because it impacted on my thought pattern and it all came down to language.

In managing obesity, participants' expressed the importance of GPs addressing the individual needs and preferences of their patient. An over‐reliance on lifestyle interventions was not perceived to be an acceptable approach for weight management, nor was an immediate offer of medication: ‘It was just straightaway, you know it's medication, go away from me with medication. I don't want medication’. Willingness to discuss the potential uptake of medication for weight management was markedly swayed in a positive direction where participants had a good interpersonal relationship with their GP. For example, where participants' describe medication as not being effective, how their GP managed the participant's expectations positively impacted upon their willingness to engage with alternative options.

[Doctors name] speaks about this medicine might suit you, but it mightn't. He tells you that going in and he's like [sic] if you come back in 3 months and tell me it doesn't work well it doesn't work and we move on and we get the next option.

Follow‐up appointments were deemed to be of high importance in the sample, however they were again perceived to vary depending on the participant's relationship with their GP. From the participant perspective, positive follow‐up appointments include the exploration of a suitable treatment approach tailored to the individual. Participants advised that follow‐up evaluation should not be led by weight loss but through an assessment of overall health and well‐being. Poor follow‐up was described as being either when it is nonexistent or where the initial prescribed treatment was deemed not suitable for the patient, and no further investigation or new treatment approach was discussed or implemented: ‘We'll see how you're getting on and if you're still struggling we'll see what else can be done. You never hear that, you just hear well, eat less, move more ‐ see you when I see you’.

I took it for a month and it worked [medication] I was like [sic] oh my god I don't really feel like eating as much as I normally would. And I went back to him to take a second set, and I took the second set and it didn't have the same impact. I remember saying it to him and he said ‘oh well that's grand so we won't do those anymore’, and that was it. There wasn't we might need to change the dose, this might not work for you, it was you aren't accepting of that drug so therefore it's not working.

## 4 Discussion

### 4.1 Principal Findings

Poor interpersonal interactions in general practice were perceived to perpetuate WS and impair the patient–provider relationship. This fracture led to reported reductions in treatment‐seeking behaviours, increased the expectation of differential healthcare, and influenced patients' trust in their GP to support them with their health concerns. Furthermore, stigmatising interactions in general practice influenced participants motivation, self‐efficacy, perceived autonomy, and intention to engage with prescribed treatments. An unexpected finding offers nuanced insight into how arbitrary lifestyle advice received in general practice was perceived to mediate internalised feelings of blame and shame, contributing to disordered eating cognitions and eating behaviours over time. The risk of cognitive distortions was amplified where general dietary advice was continually offered in the absence of investigating the participants' repeated concerns as to why traditional approaches were not successful. Unfortunately, this was not an isolated incident, but a pattern identified by many as beginning in childhood. Like obesity, development of eating disorders has a complex aetiology but onset in late childhood/early adolescence is not uncommon [43, 44]. The ways in which weight‐related health concerns are communicated to children and young people may play a major role in future weight trajectory as well as potential adoption of disordered eating behaviours.

### 4.2 Comparison With Existing Literature

Reflecting the broader literature, participants described the pervasive use of derogatory language to describe their weight and as having their presenting health concerns dismissed or attributed to weight [13, 23, 31]. In line with previous research, the frequency and intensity of stigmatising interactions experienced in general practice was perceived to be more pronounced for patients as their weight increased [45]. The options for weight management were for most, limited to oversimplified lifestyle changes perceived by participants to reflect therapeutic inertia and ambivalence. In the sample, participants reported commonly receiving generalised lifestyle advice with little to no elaboration on how to tailor it to their individual needs and preferences. There was an obvious absence of multicomponent evidence‐based behavioural interventions offered to patients in the sample, instead self‐directed diets were normalised as the prescribed approach. Inevitably weight loss attempts were difficult given the lack of personalised direction, causing internalised feelings of shame, blame and failure which influenced eating behaviours and contributed to healthcare avoidance.

Successful health gains were conflated with weight loss guided by the anthropometric measurement, BMI. The assumption of there being a normative BMI to indicate overall health was perceived to dominate decision‐making within patient–provider interactions [13]. Evidence in this study alludes to the perceived risks associated with high BMI being prioritised over evaluating the potential risk of eating pathology. This finding highlighted the layered complexity of WS when it intersects with stigma associated with disordered eating in obesity [46]. In particular, the biased assumption that disordered eating, specifically restrictive eating and binge‐purge eating behaviours are represented by ‘thinness’ and not higher weight [19]. These false attributions not only reflect the lack of understanding of the spectrum of eating disorders in general practice settings [47] but reinforce the presence of biased assumptions of the controllability of obesity and eating behaviours [46, 48].

Recent research has highlighted the significant symptomatology overlap of all eating disorders regardless of whether restrictive in nature [48]. It is also estimated that up to 50% of bariatric treatment seeking populations may have binge eating disorder, compared with 1%–2% in nonsurgical populations [49]. The present findings contextualise general practice within the current literature outlining the association of WS experienced in healthcare settings with disordered eating behaviours [12, 50]. This phenomenon is magnified where there are adverse early childhood experiences of WS in healthcare settings that persist into adulthood, as were reported by the participants in this study [15, 26]. GPs need to be made aware of the influence that historical WS can have when treating PwO [23, 51]. It is imperative that GPs do not make assumptions about eating behaviours and evaluate their patients for the risk of distorted eating cognitions and behaviours, irrespective of their patients presenting weight [19, 25, 52].

Attitudes towards the uptake of pharmacological interventions for the management of obesity were evaluated through the perceived strength of the patient–provider relationship. Similar to previous research, participants described being open to alternative methods prescribed by their GP when their expectations were positively managed, and prescribed treatments were followed‐up with an evaluation of the overall health benefits, not with a sole focus on weight loss [9, 53, 54]. This finding reflects previous research outlining patient preferences for an individualised approach to managing their obesity [13, 55]. This approach necessitates identifying contributors to obesity that are specific to the individual presentation of the patient [56]. A comprehensive evaluation of the same involves the establishment of a collaborative patient–provider relationship to identify healthcare needs and to safely support successes and setbacks [57]. Amidst calls for a better understanding of how to optimise patients' obesity‐related outcomes via available behavioural and pharmacological interventions [9], our findings highlight the need to first address stigmatising interpersonal interactions. This can be achieved by enhancing the quality of the patient–provider relationship to foster collaborative decision‐making that improves patient outcomes.

### 4.3 Reducing WS in General Practice

The effectiveness of existing WS reduction strategies remains unclear due to marginal effects and a paucity of longitudinal studies to assess long‐term impact [29]. Additionally, a crucial gap exists in understanding the perspectives of PwO on preferred solutions [32]. This study bridges a gap by offering insights from lived experience to inform future practice, and intervention design.

The findings accentuate that person‐centred communication and care is key in reducing WS within patient–provider interactions [13, 21]. Specifically, patients call for a respectful and empathetic approach to the initiation of weight‐based discourse where GPs are mindful of weight‐related terminology. There is a desired ‘considered approach’ to obesity management in which PwO feel listened to, have their health concerns taken seriously and are communicated with in a nonjudgemental manner [13]. In this study, GPs avoiding the initiation of weight‐based discussion was overwhelmingly perceived as being representative of a lack of compassion or care for the participant's well‐being. Echoing previous findings, PwO are not opposed to discussing weight, instead there is an expectation that their GP will initiate weight‐related health discussion [55, 58]. The manner in which this is imposed is the mediating factor in whether the interaction is perceived to be stigma laden.

The 5As of obesity management (Ask, Assess, Advise, Agree and Assist) is a structured interview format that facilitates the initiation of nonstigmatising person‐centred discussions about obesity in general practice [5, 59, 60]. The evidence‐based framework supports the collaborative exploration of informed and individualised treatment options for PwO [55]. This is facilitated through the guided exploration of the root drivers of obesity specific to the individual. Through an assessment of the person's history and an evaluation of potential ecological factors that may present a barrier or provide an enabler for weight management, decisions for treatment options going forward are grounded in the individual needs and preferences of the PwO, leading to better health outcomes [10].

Further education and training for GPs is recommended to encourage active participation in person‐centred care for obesity in general practice. This training should incorporate weight‐based communication training [10, 58], include updated education on the pathophysiology of obesity and, increase awareness of contemporary obesity management strategies [61]. The prescription of arbitrary lifestyle advice evaluated by reductions in BMI have been shown to cause harm by contributing to internalised WS [26, 61]. Therefore, PwO should be offered tailored, evidence‐based behavioural interventions, and supported with ongoing follow‐up appointments where obesity treatment that promotes health‐related quality of life is prioritised over weight‐loss alone [61, 62]. Finally, based on the findings in this study, in practice, it is recommended that patients are screened for disordered eating cognitions and behaviours [63, 64]. GPs are encouraged to consult and utilise the comprehensive framework outlined in the National Institute for Health and Care Excellence (NICE) guidelines to ensure best practice in the identification and assessment of potential disordered eating behaviours [65].

### 4.4 Strengths and Limitations

A strength of this study is its in‐depth reflexive qualitative approach. The research team's inclusion of personal experiences and perspectives on the phenomenon being explored enhanced reflexivity. Limitations of the study include the size and specificity of the sample to the Irish general practice context, which may not be generalisable to general practice in other countries. While the sample size is small and confined to general practice, in light of information power [35], rich, meaningful themes were generated that provide novel insights into the experiences of WS in general practice from the perspective of PwO. Despite efforts to recruit from all genders, the study is limited by the inclusion of fewer male participants. This is a common finding in WS research, reiterating the need to increase our understanding of the male experience of WS across healthcare settings. Future research should prioritise targeted recruitment of male participants to elucidate potential gender disparities and achieve a comprehensive understanding of the phenomenon.

### 4.5 Conclusions

Despite the growing body of evidence outlining the effects of WS in healthcare settings, PwO continue to experience stigmatised patient–provider interactions that impact upon their treatment seeking behaviours, eating behaviours, self‐esteem and trust in their GP. Highlighting the complexity of obesity management, our findings further reveal a perceived association between the prescription of generalised lifestyle advice, internalised WS and disordered eating behaviours. Taken together, these findings underscore the need for a paradigm shift in how obesity is supported in general practice to improve patient outcomes.

To target WS in general practice, future interventions should focus on providing weight‐based communication training underpinned by patient‐centred care and communication principles to GPs, in addition to providing updated obesity education, and increasing awareness of contemporary obesity management strategies. By prioritising respectful, collaborative, and individualised care for obesity, GPs can cultivate a supportive nonstigmatising patient–provider relationship to optimise health outcomes for people with obesity.

## Author Contributions

**Leona Ryan:** conceptualisation, methodology, investigation, writing–original draft, formal analysis. **Jane C. Walsh:** funding acquisition, methodology, review and editing, supervision. **Fiona Quigley:** formal analysis, writing–review and editing. **Michael Crotty:** conceptualisation, writing–review and editing. **Susie Birney:** conceptualisation, writing–review and editing. **Owen Conlan:** methodology, review and editing, supervision.

## Acknowledgements

We would like to extend our deepest gratitude to the participants who willingly shared their experiences with us. Your contributions will go toward improving the provision of care for obesity in general practice. L.R. is in receipt of the following financial support for the research, authorship, and/or publication of this article: This work was supported by the Science Foundation Ireland Centre for Research Training in Digitally Enhanced Reality (D‐REAL) under Grant No. 18/CRT/6224. Open access funding provided by IReL.

## Ethics Statement

Ethical approval for the study was granted by the University of Galway Research Ethics Committee (approval reference number: 2022.10.014).

## Conflicts of Interest

Michael Crotty reports honoraria for educational events or conference attendance from Novo Nordisk and Consilient Health and was a member of a Novo Nordisk advisory board. He is a member of the Irish ONCP Clinical Advisory Group and ASOI. Michael is the co‐founder and clinical lead of ‘My Best Weight Clinic’. Susie Birney reports funding to ICPO from the HSE, Novo Nordisk, and the European Coalition for People Living with Obesity (ECPO) and consulting fees or honoraria from Diabetes Ireland, ECPO, and Novo Nordisk. Susie is the Executive Director of ICPO and the Secretary of ECPO. The other authors declare no conflicts of interest.

## Supporting information

Supporting information.

## Data Availability

The data that support the findings of this study are available on request from the corresponding author. The data are not publicly available due to privacy or ethical restrictions.
