# Supplementary material for: ‘Beyond the Scale’: A Qualitative Exploration of the Impact of Weight Stigma Experienced by Patients With Obesity in General Practice
Source: Health Expect. 2024 Jun 11;27(3):e14098. doi: 10.1111/hex.14098 (PMC11165259; doi:10.1111/hex.14098)
Supplement: Supplementary file 1 — Supporting information. [file HEX-27-e14098-s001.docx]

| **Greeting and introduction** | - Thank you so much for agreeing to participate in this interview today! Your insights are valuable to us. Before we start, I’d like to go over a few points to make you aware of the process and to give you time to ask me about anything that you may be wondering about. - My name is XX and I'm conducting this interview as part of a research project focusing on weight stigma in general practice settings. - As mentioned in the information sheet, we're interested in learning about your perceptions on and experiences of weight stigma in this setting. - Throughout the interview, please feel free to skip any questions that make you uncomfortable. Your participation is completely voluntary. - We will record our conversation for accurate transcription. However, your identity will be protected completely. We will remove your name and any identifying details, like specific locations, so no one will be able to recognise you even if they read the transcript. - Please don't hesitate to let me know if you want to stop the recording at any point. - Do you have any questions before we begin? |
| --- | --- |
| **Interview Topic Guide** | **Overarching Question(s)** |
| Weight stigma experiences in general practice settings | How would you describe your experience of weight stigma in general practice? |
| The presentation of weight stigma in the patient-provider realtionship | How would you describe the quality of the relationship you have with your GP? In your opinion, does weight stigma influence it, if so, how? |
| The impact of weight stigma on the delivery of care | Have you had any health concerns not listened to or blamed on your weight?  Have you been refused care based on your weight? |
| The impact of weight stigma on patients’ engagement with healthcare services | Has having these experiences ever put you off wanting to visit with any healthcare services? |
| Suggestions for reducing weight stigma within the patient provider relationship. | In your opinion, what could GPs do in their practice to help reduce patients experiencing weight stigma? |
| **Closing the interview** | Is there anything else that you would like to add?  Thank you for taking time to contribute to this research, I really appreciate you sharing your experiences with me. |

**Supplementary Material**

**Table S1: *Qualitative Interview Protocol***
